# Implantation of sigmoid colon cancer into the endoscopic resection site of intramucosal rectal cancer: A case report

**DOI:** 10.1002/deo2.193

**Published:** 2022-12-08

**Authors:** Kyohei Nishino, Masanori Hongo, Naoko Mori, Kazumi Shimamoto, Yu Kobayashi, Fumiyasu Nakamura, Michiko Hino, Takeshi Togawa, Akira Andoh, Hiromitsu Ban

**Affiliations:** ^1^ Department of Gastroenterology Omi Medical Center Shiga Japan; ^2^ Department of Pathology Omi Medical Center Shiga Japan; ^3^ Department of Digestive Surgery Omi Medical Center Shiga Japan; ^4^ Department of Medicine Shiga University of Medical Science Shiga Japan

**Keywords:** colorectal neoplasms, endoscopic submucosal dissection, implantation, *KRAS*, local recurrence

## Abstract

A 70‐year‐old woman was diagnosed with intramucosal rectal cancer and advanced sigmoid colon cancer at the same time. First, the intramucosal rectal cancer was curatively resected by endoscopic submucosal dissection, and surgery was subsequently performed for sigmoid colon cancer. After 20 months, a follow‐up colonoscopy revealed a tumor growth at the ulcer scar of the endoscopic submucosal dissection. Histological findings and *KRAS* mutation analysis suggested implantation of sigmoid colon cancer to the post‐endoscopic submucosal dissection site of intramucosal rectal cancer.

## INTRODUCTION

In the surgical field, implantation of exfoliated cancer cells has been recognized as one of the mechanisms contributing to tumor recurrence at the suture site after surgery for colorectal cancer.^1^, ^2^ Exfoliated cells are considered to colonize and proliferate in tissue exposed by surgery.^1^, ^2^, ^3^ Endoscopic treatment, such as endoscopic submucosal dissection (ESD), is widely performed as a treatment option for superficial neoplasm of the gastrointestinal tract.^4^ Patients with synchronous multiple colorectal cancers sometimes need a combination of endoscopic and surgical treatments, although endoscopic treatment in the presence of advanced colorectal cancer carries a risk of cancer cell implantation at the site of endoscopic resection.^5^ However, there are a few reports describing a tumor recurrence due to the implantation of primary tumor cells into the post‐endoscopic ulcer.

Here, we report a case of cancer recurrence due to the implantation of sigmoid cancer into the post‐ESD ulcer of intramucosal rectal cancer. Implantation of sigmoid cancer was strongly supported by an identical *KRAS* mutation in the recurrent lesion.

## CASE REPORT

**FIGURE 1:**
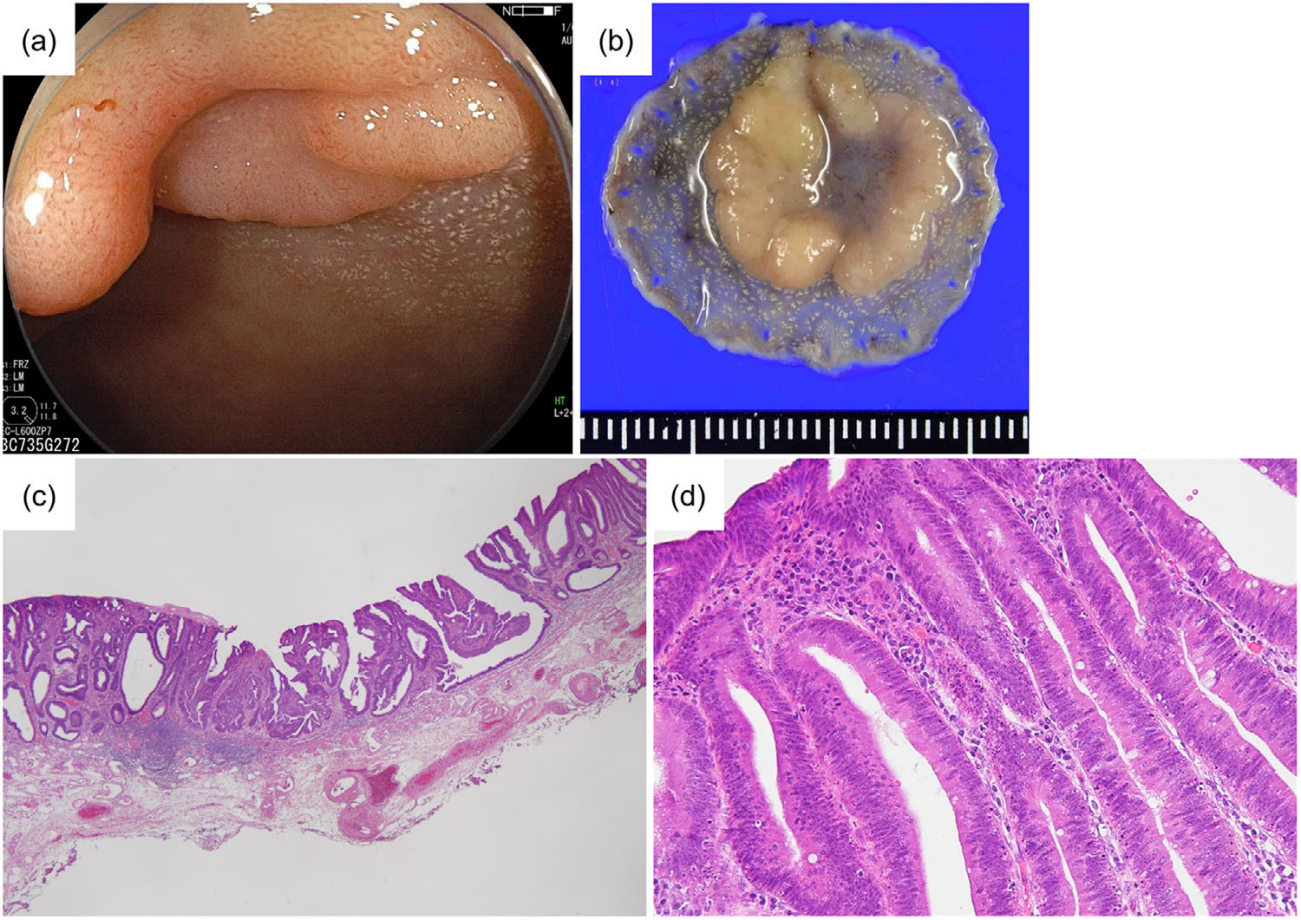
Intramucosal rectal cancer resected by endoscopic mucosal dissection. (a) Endoscopic finding of an elevated lesion with central depression. (b) Resected specimen (20×19 mm). (c,d) Histopathological examination revealed an intramucosal well‐differentiated adenocarcinoma. Original magnification: (c) ×20, (d) ×200

**FIGURE 2:**
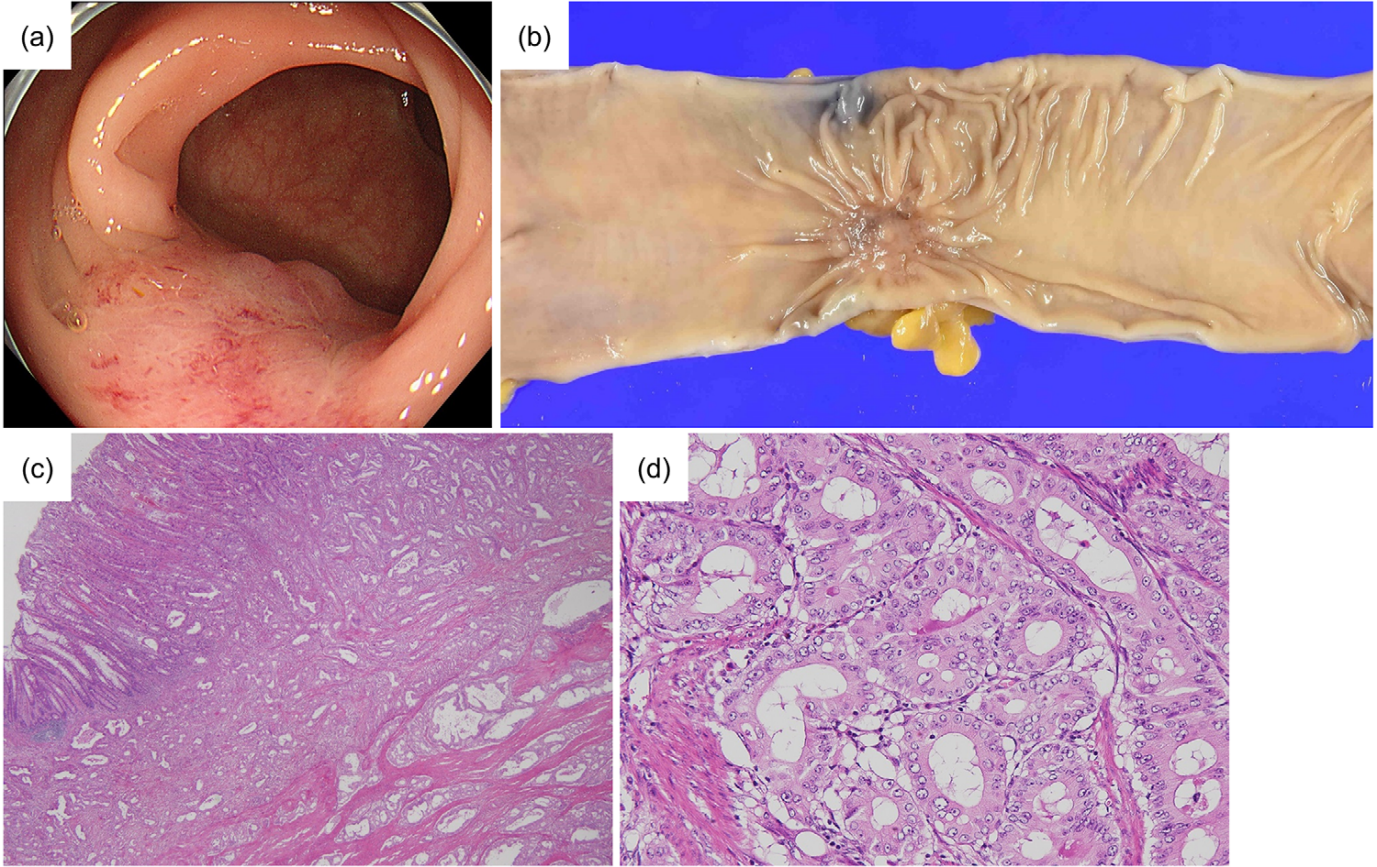
Sigmoid colon cancer resected by surgery. (a) Endoscopic finding. Elevated lesion with redness and irregular surface occupying one‐third of the circumference. (b) The resected specimen of a 17 × 22 mm tumor with fold convergence. (c, d) Histopathological examination revealed a moderately to poorly differentiated adenocarcinoma with serosal invasion. Original magnification: (c) ×20, (d) ×200

A 70‐year‐old woman was referred to our hospital for the treatment of a rectal tumor located 10 cm from the anal verge and advanced sigmoid colon cancer located 20 cm from the anal verge. The rectal tumor was endoscopically diagnosed as intramucosal rectal cancer (0–IIa+IIc) and an en bloc resection was performed by ESD in advance (Figure 1a). The resected specimen was 20 × 19 mm in diameter and its lateral margin was negative (Figure 1b). Histopathological examination revealed an intramucosal well‐differentiated adenocarcinoma with negative horizontal and vertical margins and no lymphovascular invasion (Figure 1c,d). The procedure was considered a curative resection. After two months, a laparoscopic high anterior resection with lymph node dissection was performed for sigmoid colon cancer (Figure 2a,b). Histopathological examination revealed a moderately to poorly differentiated adenocarcinoma (pT4a N1 M0; Figure 2c,d). Postoperative adjuvant chemotherapy was administered for six months.

**FIGURE 3:**
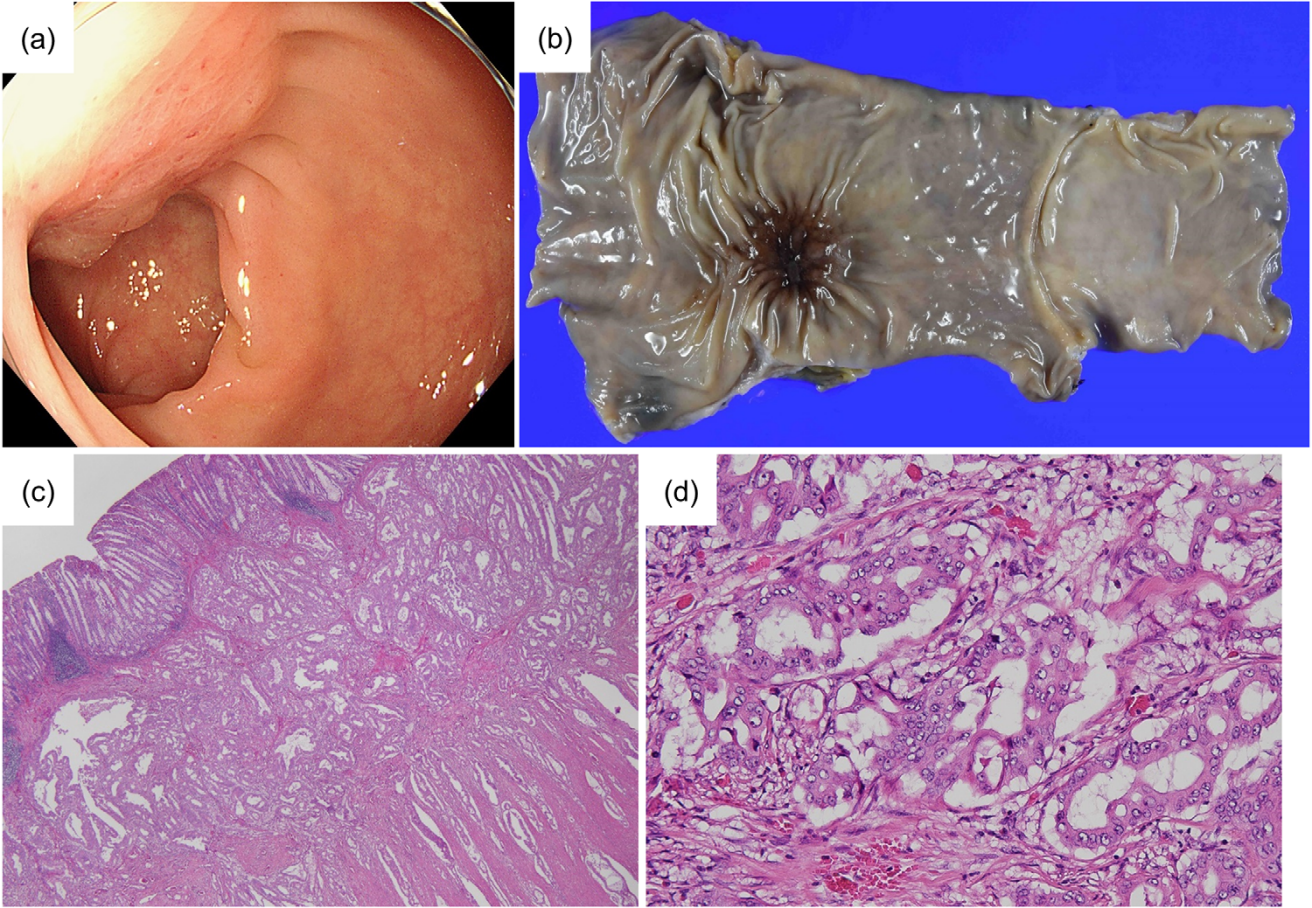
Recurrent lesion at the post‐endoscopic mucosal dissection site. (a) Colonoscopy showing a submucosal tumor‐like lesion at the post‐endoscopic mucosal dissection scar. (b) The resected specimen showing a 33 × 25 mm tumor with central depression. (c,d) Histopathological examination revealing a moderately differentiated adenocarcinoma grown mainly in the submucosa and muscularis propria. The histology was similar to the previously resected sigmoid colon cancer. Original magnification: (c) ×20, (d) ×200

Twenty months after surgery, a computed tomography scan showed a thickening of the rectal wall, and a colonoscopy revealed a submucosal tumor‐like lesion at the post‐ESD scar (Figure 3a). Local recurrence was suspected by histological examination of the biopsy specimen. A laparoscopic low anterior resection was performed (Figure 3b). Histopathological examination revealed a moderately‐differentiated adenocarcinoma (pT3N1M0; Figure 3c,d), which was similar to that of the previously resected sigmoid colon cancer.

*KRAS* mutation analysis indicated that the intramucosal rectal cancer was positive for p.G13D, and both the primary sigmoid cancer and recurrent cancer at the ESD scar were positive for p.G12V. These results strongly suggested that the recurrent lesion was a cancer cell implantation from sigmoid colon cancer.

## DISCUSSION

We reported a case of implantation of sigmoid colon cancer into the post‐ESD site of intramucosal rectal cancer. An identical *KRAS* p.G12V mutation in both the primary sigmoid cancer and the recurrent lesion confirmed the cancer cell implantation.

Umpleby et al. previously demonstrated the presence of viable exfoliated cancer cells in the lumen of patients with advanced colorectal cancer.^1^ In colorectal cancer surgery, these cells are considered to be associated with cancer cell implantation in an anastomotic suture line.^1^, ^6^ Similarly, such a phenomenon can be expected in ESD in the presence of colorectal cancer.

There are a few reports of tumor cell implantation associated with endoscopic resection. Tajika et al. reported a case of cancer cell implantation from rectosigmoid colon cancer into the endoscopic mucosal resection (EMR) site of a synchronous rectal carcinoid.^5^ Similar to our case, they initially performed EMR for the carcinoid in the presence of advanced sigmoid colon cancer. Nakano et al. reported a case of recurrent rectal adenoma which was suspected to have been induced by tumor cell implantation during an ESD procedure.^7^ This suggests that the ESD procedure may cause the exfoliation of tumor cells, leading to tumor cell implantation in the post‐ESD ulcer. In our case, the rectal lesion was curatively resected by ESD, and the histology of the locally recurrent lesion was similar to that of sigmoid colon cancer. The *KRAS* gene mutation in the recurrent lesion was consistent with sigmoid colon cancer but differed from rectal cancer. These findings strongly suggested cancer cell implantation from the sigmoid colon cancer to the ESD site.

Various factors may influence the establishment of tumor cell implantation into the endoscopically resected site. Maeda et al. have proposed two possible factors involved in cancer cell implantation: the presentation of viable cancer cells and the susceptibility of damaged tissue to colonization and proliferation of cancer cells.^6^ The number of viable cancer cells reaching the damaged mucosa may be related to the size of the original tumor. It can be easily envisaged that a larger, more advanced cancer would serve more viable cells to the damaged mucosa. Certainly, in our case and that of Tajika et al., endoscopic resection was performed in the presence of advanced sigmoid cancer, which was located close to the endoscopic resection site.^5^ On the other hand, factors governing tissue susceptibility remain unknown. It can therefore be considered that larger post‐resection ulcers have an increased potential for cancer cell colonization, although some previous studies demonstrated that cancer cell implantation developed in a small post‐resection ulcer of only a few millimeters.^5^, ^8^ Considering bowel peristalsis and fecal movement, the proximal location of advanced cancer may cause a sustained supply of viable cancer cells. So, in some cases, it may be preferable to perform surgical resection of the major lesion with priority over endoscopic resection of small non‐urgent lesions. It may be beneficial to complete the closure of endoscopic resection sites if endoscopic resection is necessary prior to surgery. As reported by Inoue et al., intraluminal lavage after ESD may be also effective to remove exfoliated tumor cells.^9^

The malignant potential of cancer cells may be one of the risk factors for cancer cell implantation but there are few reports on this matter. In this study, we detected a *KRAS* p.G12V mutation in both sigmoid cancer and recurrent lesion. *KRAS* mutation in codon 12 has been reported to confer increased oncogenic potential of cancer cells when compared to the codon 13 mutation,^10^ leading to poor prognosis and resistance to anti‐EGFR therapy. Tajika et al. also reported a *KRAS* p.Q61R mutation in both the primary and recurrent lesions of possible cancer cell implantation,^5^ but the biological and clinical influences of this mutation remain unclear. In the future, the biological background related to cancer cell implantation needs to be clarified by the accumulation of more cases. For this purpose, *KRAS* mutation analysis may be a good biomarker.

In conclusion, endoscopists need to pay attention to the risk of tumor cell implantation when performing endoscopic resection of a colorectal tumor in the presence of advanced colorectal cancer. Analysis of the *KRAS* mutation may be helpful for the diagnosis of cancer cell implantation.

## CONFLICT OF INTEREST

None.
